# Ionizing Radiation, Antioxidant Response and Oxidative Damage: Radiomodulators

**DOI:** 10.3390/antiox12061219

**Published:** 2023-06-05

**Authors:** Elena Obrador, Alegría Montoro

**Affiliations:** 1 Elena Obrador Department of Physiology, Faculty of Medicine and Odontology, University of Valencia, 46010 Valencia, Spain; 2 Alegría Montoro, Radiation Protection Service, University and Polytechnic Hospital La Fe, 46021 Valencia, Spain; almonpas@hotmail.com

Ionizing radiation (IR) is the energy released by atoms in the form of electromagnetic waves (e.g., X or gamma rays) or particle radiation (alpha, beta, electrons, protons, neutrons, mesons, prions, and heavy ions) with sufficient energy to ionize atoms or molecules [1]. IR induces DNA breaks leading to direct molecular damages or even cell death, whereas in the case of cell survival, it can lead to carcinogenesis or other abnormalities [2,3]. The highly reactive free radicals formed by the radiolysis of water (superoxide anion (O_2_^•−^), hydroxyl radical (•OH), hydrated electron (e^−^_(aq)_), and hydrogen peroxide (H_2_O_2_)) and the subsequent oxidative stress are the main cause of most of the molecular (DNA, lipids, proteins, etc.) damages [1,4]. In turn, cells injured by IR are responsible for inducing radiation bystander effects (RIBEs) in non-exposed tissues, manifested by changes including (but not limited to) gene expression alterations, chromosomal aberrations, micronuclei formation, secretion of miRNAs and or exosomes, and cell death/proliferation and/or transformation. In that sense, overproduced reactive oxygen species (ROS) and reactive nitrogen species (RNS) are considered initiators, whereas nitric oxide, the transforming growth factor beta (TGF-β), and other inflammatory cytokines are considered effectors involved in RIBE [5].

Radiation-induced biological effects are determined by the type of radiation, dose rate and total dose received, fractionation and protraction, penetration capacity, linear energy transfer, cell or tissue affected, and time of exposure [6]. The survival response can be modulated by different factors, e.g., DNA repair mechanisms, antioxidant defences, intrinsic radiosensibility of tissues, inflammatory response, RIBE, health condition, and/or the administration of radiomodulators. Radiomodulators include radioprotectors, radiomitigators, and molecules with radiosensitizing properties. The first ones reduce or prevent the damage induced by IR (generally acting as chelators of free radicals, antioxidants, …), but to be effective, they must be administered before or at the same time as exposure [2]. In contrast, radiomitigators help to recover or attenuate radio-induced damages even when they are administered after radiation exposition [3,7], which represents an important strategic advantage. Mechanisms involved in the radiomitigating activity include the restoration of the cellular antioxidant defence mechanisms, the prevention of excessive inflammatory responses, and/or the repair of damaged tissues [5]. Finally, radiosensitizers can increase radio-induced damage in cells or tissues, and their use is mainly focused on cancer treatment, where it is especially important to induce cytotoxicity in the cancer cells without affecting healthy tissues [8,9,10].

Nuclear technology is becoming widely used in many diverse fields, i.e., industry, medicine, military, and basic research. Although a few years ago, exposure to IR was restricted to accidents at nuclear power plants or terrorist attacks, nowadays, the risk is increased by the improper and careless management of IR in the radiographic industry and in radiation oncology [11]. Moreover, IR exposure is unavoidable in the case of cancer patients undergoing radiotherapy, and it will also be unavoidable for future aerospace ship crews [12]. All this raises the inexorable need to develop strategies that may allow rapid interventions to prevent or reduce radiation-induced damages. Significant advances have been made in public health and the clinical planning intended to prevent or improve the response to an accidental exposure [5]. However, despite the strong economic and scientific efforts over recent decades, at present, clinical biomarkers of radiation-induced damage and drugs that can effectively protect against lethal IRs are still an unmet need.

Overall, this Special Issue provides the readers with an understanding of the latest advancements in the bioefficacy and/or mechanisms of action of several promising radiomodulators [2,3,5,7,9,10,13,14,15,16], the capacity of hyperthermia to increase cancer treatment efficacy [17], a new clinical model to evaluate the radio-induced damages [15,18], and novel IR biomarker technologies [19,20].

N-acetyl-5-methoxytryptamine (melatonin), often referred to as a “sleep hormone”, is produced by the pineal gland. Its free radical scavenger and antioxidant actions contribute to reducing nitric oxide formation, which facilitates the decrease in the inflammatory response after IR exposition [5]. According to the experiments concerning whole body irradiation, melatonin administered both before and after IR exposure increased the survival rate of examined animals, reduced symptoms of acute irradiation syndrome, and improved radio-induced oxidative stress and histopathological damages [21]. Animal studies have confirmed that melatonin can also alleviate radiation-induced cell death via inhibition of proapoptotic genes (e.g., Bax) and upregulation of antiapoptotic genes (e.g., Bcl-2) [22]. The radioprotective and radiomitigating efficacy of melatonin was confirmed by Abdullaev et al. in tissues (brain and spleen) that exhibit different proliferative activity and radiosensitivity [2].

Peroxiredoxins are capable of reducing a wide range of inorganic and organic peroxides. Novoselova et al. show that peroxiredoxin 6 treatment normalized p53 and NF-κB/p65 expression, p21 levels, DNA repair-associated genes, TLR expression, pro-inflammatory cytokine production (TNF-α and IL-6), and apoptosis in irradiated 3T3 cells cultures. Moreover, the IR-induced suppression of the Nrf2 activity (a key regulator of the antioxidant cellular response) could be restored by peroxiredoxin 6, even when it was administered after radiation exposure [3].

Wagle et al. evidenced that supplemented ferulic acid attenuates the total body irradiation-mediated bone marrow damages, stem cell senescence, and hematopoietic injury by enhancing the antioxidant defence. Thus pointing out its potential as a radioprotective countermeasure [13].

Lactoferrin is a multifunctional glycoprotein present in mammalian secretory fluids and in neutrophil granules. The study of Kopaeva et al. provided evidence for the radiomitigating potential of human Lactoferrin on mice subjected to sublethal irradiation. Their results show that treated mice increased their survival from 28% to 78%, attenuated weight loss, normalized their behaviour, and also increased the leukocyte account, serum homeostasis parameters, and structural organization of the spleen [7].

Pelvic irradiation-induced mucositis secondarily leads to changes in the composition of microbiota and reduces its diversity, all factors that contribute to radiation enteropathy [23,24]. Microbiome dysbiosis contributes to the overexpression of proinflammatory cytokines and weakens the function of the intestinal epithelial barrier [24]. Typical symptoms of gastrointestinal radiation injury, including vomiting, anorexia, abdominal pain, and diarrhoea, can seriously affects patients’ quality of life after or during radiotherapy treatment [25,26]. Limiting the intestinal irradiation dose and using a lower fractionated dose helps to relieve symptoms, but compromises and reduces the anti-tumour efficacy of the treatment [27]. Probiotics can attenuate radiation-derived enteritis side effects by restoring microbiota, regulating the immune system, downregulating proinflammatory cytokines, regulating apoptosis, and reversing ecological dysregulation [28,29]. Thus, the development of probiotic therapy to prevent the radiotherapy-induced diarrhoea is a hot topic, but with contradictory efficacy according to several recent studies [30,31,32]. Although some studies have proposed that *Lactobacillus rhamnosus* supplementation is efficacious for preventing radio-induced enteritis [33,34], Segers et al.’s results [14] evidence that, although supplementation with *Limnospira indica* PCC 8005 or *Lacticaseibacillus rhamnosus* GG ATCC prevented radio-induced dysbiosis, probiotic administration did not preserve the intestinal protective barrier or did not have significant immunomodulatory effects.

Radiotherapy failure and poor tumour prognosis are primarily attributed to radioresistance. In 1924, Otto Warburg observed that tumours consumed large quantities of glucose while secreting high levels of lactate, irrespective of the tissue oxygen concentration [35]. Since then, this “aerobic glycolysis” has been shown to benefit tumour growth by providing intermediates needed to maintain high rates of cellular division. Moreover, the link between glucose metabolism and redox stress is relevant for the cancer radiation response. Bian et al. present an interesting review exposing the mechanisms involved in mitochondrial metabolism and cancer radioresistance, targeting mitochondrial signalling pathways to reverse radiation insensitivity [36].

Planarians are invertebrate flatworms with stem cells constantly replacing old, damaged, or dying cells. Combined with new genomic technologies, the planarian sensitivity to IR provides an outstanding tool for the evaluation of potential radioprotective agents [18]. In this, Tsarkova et al.’s study on the planarian model confirms the antioxidant properties of Tameron against X-ray-induced and menadione-induced oxidative stress [15].

In cancer therapeutics, the development of radiosensitizers would represent a great advance, especially if treatment can also exert radioprotective effects on healthy tissues. Pacifico et al. evidence that a polyphenol-rich Olea europaea L. cv. Caiazzana Leaf extract mitigates radiation-induced DNA damage in normal cell lines, whereas it exacerbates radiation damage in cancer cells [9].

The prognosis of patients diagnosed with pancreatic cancer remains dismal, with a less than 3% survival at 5 years [37]. At supraphysiologic levels, ascorbate (vitamin C) acts as a pro-oxidant, donating electrons to form H_2_O_2_ and, thereby, causing selective cytotoxicity and oxidative stress in pancreatic cancer cells [38,39]. Thus, pharmacological ascorbate has potential as a radiosensitizer at the same time that several studies evidence its radioprotective potential in normal tissues [40]. During recent years, Auranofin has garnered interest for its anticancer potential, although its mechanisms of action are not completely elucidated. Steers et al. review the mechanism and effectiveness of auranofin alone and in combination with ascorbate and IR, in the treatment of pancreatic cancer [10].

Applying focused hyperthermia (HT), the temperature of the affected area is raised to around 40–45 °C using different methods, i.e., microwave, radiofrequency, ultrasound, or infrared radiations. In cancer therapy, a specific area is exposed to high temperatures to kill cancer cells or make them more sensitive to other cancer treatments, such as chemo and/or radiotherapy [17,41,42]. The tumour microenvironment is characterized by nutrient deprivation, limited oxygenation, and highly acidic conditions, properties associated with low effects on the tumour response to radiation [42]. HT increases tumour perfusion and re-oxygenation, drug uptake, and ROS production, inhibits the repair of radiation-induced damage, and induces cancer cell apoptosis [43,44]. In addition to the various thermoradiobiological effects, it has recently been shown that HT has immunostimulatory effects involving the innate and adaptive immune systems, thereby inducing systemic anti-tumour immune responses [45]. Kwon et al. review the latest advances regarding the efficacy of HT in the treatment of cancer and its possible synergistic effects with radiotherapy [17].

Sagkrioti et al. developed a database termed RadBioBase, which includes comprehensive transcriptomes of mammalian cells across healthy and non-healthy tissues when responding to a range of radiation types and doses. Their results evidence that the effects of high linear energy transfer (LET) radiation on cell transcriptomes significantly differ from those caused by low LET and, importantly, are consistent with immunomodulation, inflammation, oxidative stress responses, and cell death. The transcriptome changes also depend on the dose, since low doses up to 0.5 Gy mainly involve cytokine cascades, while higher doses are mainly linked to ROS metabolism. Overall, their results suggest that different radiation types and doses can trigger distinct cell-intrinsic and cell-extrinsic pathways, which may facilitate manipulation to improving radiotherapy efficiency and reducing systemic toxicities [19].

The serum proteomic and oxidative modification profiling in mice exposed to total body X-Irradiation evidenced a dose-dependent and oxidation-related response involving six different serum proteins (angiotensinogen, odorant-binding protein 1a, serine protease inhibitor A3K, serum paraoxonase/arylesterase 1, prothrombin, and the epidermal growth factor receptor). Consequently, Yamaguchi et al. suggest the possible application of these changes as novel biomarkers to validate the radiation dose [20].

Electron radiation is widely used to ensure the microbiological safety of food and drugs. One of the most important advantages of this method is that it is carried out at low temperatures, which is important for heat-sensitive products. Many brands of nutraceuticals containing resveratrol are available, and Rosiak et al. show that, far from endangering the biological properties of resveratrol, electron beam radiation (even at a dose of 25 kGy) can increase the antioxidant properties of resveratrol [16].

Finally, Obrador et al. discuss the best choices for triage, dose assessment, and victim classification in the case of large-scale atomic or radiological events. Furthermore, our review focuses on the available medical countermeasures (radioprotectors, radiomitigators, radionuclide scavengers) that can implement the response to an accidental radiation exposure or can help to increase survival in cancer patients [5].

As a whole, these 11 research [2,3,7,9,13,14,15,16,18,19,20] and 4 review articles [5,10,17,36] from leading experts bring to the field exciting discussions on different aspects, new findings, and new perspectives, thus making this Special Issue an essential read for anyone interested in the impact of radiation exposure on human health.
